# A scalability study of phylogenetic network inference methods using empirical datasets and simulations involving a single reticulation

**DOI:** 10.1186/s12859-016-1277-1

**Published:** 2016-10-13

**Authors:** Hussein A. Hejase, Kevin J. Liu

**Affiliations:** Department of Computer Science and Engineering, Michigan State University, 428 S. Shaw Lane, East Lansing, MI USA

**Keywords:** Phylogenetic network, Phylogenetic inference, Phylogenomics, Phylogenetics, Scalability, Large-scale, Incomplete lineage sorting, Gene flow, Mutation, Performance study, Mouse

## Abstract

**Background:**

Branching events in phylogenetic trees reflect bifurcating and/or multifurcating speciation and splitting events. In the presence of gene flow, a phylogeny cannot be described by a tree but is instead a directed acyclic graph known as a phylogenetic network. Both phylogenetic trees and networks are typically reconstructed using computational analysis of multi-locus sequence data. The advent of high-throughput sequencing technologies has brought about two main scalability challenges: (1) dataset size in terms of the number of taxa and (2) the evolutionary divergence of the taxa in a study. The impact of both dimensions of scale on phylogenetic tree inference has been well characterized by recent studies; in contrast, the scalability limits of phylogenetic network inference methods are largely unknown.

**Results:**

In this study, we quantify the performance of state-of-the-art phylogenetic network inference methods on large-scale datasets using empirical data sampled from natural mouse populations and a range of simulations using model phylogenies with a single reticulation. We find that, as in the case of phylogenetic tree inference, the performance of leading network inference methods is negatively impacted by both dimensions of dataset scale. In general, we found that topological accuracy degrades as the number of taxa increases; a similar effect was observed with increased sequence mutation rate. The most accurate methods were probabilistic inference methods which maximize either likelihood under coalescent-based models or pseudo-likelihood approximations to the model likelihood. The improved accuracy obtained with probabilistic inference methods comes at a computational cost in terms of runtime and main memory usage, which become prohibitive as dataset size grows past twenty-five taxa. None of the probabilistic methods completed analyses of datasets with 30 taxa or more after many weeks of CPU runtime.

**Conclusions:**

We conclude that the state of the art of phylogenetic network inference lags well behind the scope of current phylogenomic studies. New algorithmic development is critically needed to address this methodological gap.

**Electronic supplementary material:**

The online version of this article (doi:10.1186/s12859-016-1277-1) contains supplementary material, which is available to authorized users.

## Background

In recent studies, gene flow – the process by which genetic material is exchanged between different populations and/or species existing at the same point in time – has been shown to have played a major role in the evolution of a diverse array of metazoans, including humans and ancient hominins [1, 2], mice [3], and butterflies [4]. Each of these organisms (as well as many others across the Tree of Life [5–7]) has a phylogeny, or evolutionary history, which cannot be represented as a tree, where a branching event reflects strict bifurcating and/or multifurcating speciation/splitting and subsequent genetic isolation of the resulting species/populations. In these cases, the phylogeny takes the more general form of a directed acyclic graph known as a phylogenetic network [8]. Phylogenetic networks are categorized as either explicit or implicit networks. Reticulations in explicit networks are ascribed to specific evolutionary processes (e.g., gene flow). In contrast, graphical structure in an implicit network summarizes conflicting phylogenetic signal but lacks a specific biological interpretation. For this reason, we focus our attention on explicit phylogenetic networks and we hereafter omit the “explicit” qualifier for brevity.

Similar to phylogenetic trees, phylogenetic networks are typically inferred using computational analyses of multi-locus biomolecular sequence data. The most widely used approach is a concatenation analysis which estimates a single phylogeny for all loci [9]. In the context of species tree inference, methods used for concatenation analysis typically only account for sequence mutation [10]. Representative examples of concatenation-based network inference methods include Neighbor-Net [11] and the least squares method of Schliep [12], which we refer to here as SplitsNet. A primary complication with the concatenation approach is that different loci in a genome commonly exhibit local genealogical incongruence (i.e., gene trees can differ from each other and the species phylogeny in terms of topology and/or branch length) due to the complex interplay of different evolutionary processes that shaped the genomes. These include gene flow, sequence mutation, gene duplication and loss, recombination, and incomplete lineage sorting (ILS). ILS occurs when lineages from two genetically isolated populations coalesce at a time more ancient than their most recent common ancestral population, and is known to play a crucial role in the evolution of much of the Tree of Life [9]. Under neutral evolution, genetic drift – the outcome of purely stochastic inheritance over successive populations – can cause ILS; other factors contributing to the maintenance of ancestral polymorphisms and ILS include balancing selection.

In contrast to concatenation analysis, multi-locus methods infer species phylogenies in the presence of these evolutionary processes acting in combination. The most widely used multi-locus methods perform inference that account for a broad set of evolutionary processes, including sequence mutation, gene flow, and ILS [13–15]. Multi-locus methods are broadly classified by whether or not they impose the requirement that a phylogenetic hypothesis be specified a priori.

The main focus of our study is the category of methods that perform full inference by searching among all possible phylogenies defined on a set of taxa. Many of these methods utilize a gene-tree/species-phylogeny reconciliation approach (or summary approach), where local trees estimated from different loci – referred to as gene trees – are used as input rather than sequence alignments from the loci [16–19]. The full inference procedure therefore requires two phases: a first phase where a set of gene trees is estimated from biomolecular sequence alignments, and a second phase where the gene trees are used to estimate a species phylogeny. The multi-locus methods are further classified by the optimization criterion used for inference. Earlier parsimony-based approaches (e.g., the method of Yu et al. [20], which we refer to here as MP, which stands for maximum parsimony) utilize the minimize deep coalescence (MDC) criterion proposed by Maddison [8], which seeks the species phylogeny that minimizes the number of deep coalescences necessary to explain a given set of gene trees. More recently, probabilistic approaches perform phylogenetic network inference under an explicit evolutionary model that combines the coalescent model with biomolecular substitution models. Examples include two different methods proposed by Yu et al. [21] that are implemented in the PhyloNet software package [22], which differ primarily in their use of branch length information: one method uses the approach of Degnan and Salter [23] to calculate model likelihood using only gene tree topologies, and the other method substitutes an alternative approach to calculate model likelihood using gene tree topologies and branch lengths. We therefore refer to these methods as MLE (which stands for maximum likelihood estimation) and MLE-length, respectively. These probabilistic approaches have been noted to have high computational requirements, and model likelihood calculations were found to be a major performance bottleneck [24]. For this reason, pseudo-likelihood approximations to full model likelihood calculations have been proposed, including the method of Yu et al. [24] (referred to here as MPL, which stands for maximum pseudo-likelihood), which substitutes pseudo-likelihoods in the optimization criterion used by MLE, and SNaQ (Species Networks applying Quartets) [25], which combines the use of pseudo-likelihoods under a coalescent-based model with quartet-based concordance analysis [26]. As suggested by Yu et al. [14], the techniques used by Bryant et al. [27] to infer a species tree directly from an input sequence alignment – effectively integrating over gene tree distributions at different loci – would provide an alternative to reconciliation-based species network inference, but scalable inference methods using this alternative approach have yet to be proposed and remain for future work as of this writing; preliminary experiments by Yu et al. [14] suggest that the scalability challenges of this approach will be greater than with state-of-the-art reconciliation-based approaches. All of these multi-locus methods address problems that are either known or suspected to be NP-hard [14, 15]. For this reason, heuristics are necessary to enable efficient inference under the optimization criteria associated with these methods. The practical design of the heuristics are essential to accuracy and computational efficiency.

A second category of methods requires a phylogenetic hypotheses to be provided a priori. Methods in this category are typically used to address high-level questions such as detecting gene flow (e.g., the D-statistic [1] and its extensions [13]), inferring ancestral population sizes and other population genetic quantities (e.g., the CoalHMM method [28] which utilizes a hidden Markov model (HMM) to capture within-genome sequence dependence), and detecting genomic loci involved in gene flow (e.g., PhyloNet-HMM [29]). We note that these inference problems are contained within the general phylogenetic inference problem. Since the primary focus of our study is the general phylogenetic network inference problem rather than special cases thereof, we do not consider these methods further.

Thanks to rapid advances in genome sequencing and related biotechnologies [30], large-scale phylogenetic studies involving many dozens of genomes or more are now common (see [31] for a survey). These developments pose two primary scalability challenges: (1) the number of taxa in a study, and (2) sequence divergence, which reflects the evolutionary divergence of the taxa in a study.

For the special case of phylogenetic tree inference from phylogenomic data, recent studies have examined these scalability challenges [17, 32, 33] (including evolutionary scenarios involving gene flow [34, 35]) and proposed new methods for large-scale analysis [32, 36, 37]. In contrast, for the more general case of phylogenetic network inference, the limits of scalability on inputs with more than a few dozen taxa as well as performance at these limits have yet to be established. What are the computational requirements of state-of-the-art methods, and what is their accuracy on large-scale inputs with dozens of taxa or more?

To resolve these open questions, we conducted a scalability study of state-of-the-art phylogenetic network inference methods on both simulated and empirical datasets. To our knowledge, our study is the first to address these open questions. We chose representative methods from the different categories discussed above: Neighbor-Net and SplitsNet (from the category of concatenation methods), MP (from the category of parsimony-based multi-locus inference methods), MLE and MLE-length (from the category of probabilistic multi-locus inference methods that use full likelihood calculations), and MPL and SNaQ (from the category of probabilistic multi-locus inference methods that use pseudo-likelihood approximations to the full model likelihood). Following the practice of prior simulation studies [14, 25], our performance comparison focuses on the simpler case of search among phylogenetic networks with the correct number of reticulations. The more general case of search among network hypotheses with differing numbers of reticulations necessitates the use of model selection techniques to balance model fit versus complexity, and is suspected to be more difficult for this reason [14, 38]. Furthermore, our performance study focuses on a constrained class of phylogenetic networks with at most a single reticulation, which are a subset of other widely studied classes of phylogenetic networks (e.g., galled trees [39, 40]); the undirected version of a phylogenetic network with one reticulation (which is obtained by ignoring edge directionality) is referred to as a unicyclic network [41]. Our findings therefore provide a bound on the performance of the methods in our study, since more complex networks are anticipated to present even greater scalability challenges [14]. Our performance study utilized empirical data from past studies of natural mouse populations and synthetic data which were simulated to capture a range of evolutionary scenarios involving a single reticulation. The performance of the phylogenetic network inference methods on the empirical and synthetic data was evaluated using three performance measures: (1) computational time, (2) memory usage, and (3) topological accuracy.

## Methods

### Simulation study

**Generation of random model networks.** The random model networks used in our simulation study were generated by first sampling random model trees using r8s version 1.7 [42]. The following script was used to simulate random birth-death model trees for 5, 6, 7, 9, 10, 15, 20, 25, and 30 taxa:



Using a custom script, the branches of each random model tree were scaled to obtain height *h*. We examined two different *h* settings: a height of 1 was used throughout the study, except for experiments involving inferred gene trees where a height of 5 was used. This range of heights correspond to moderate to high levels of ILS based on the classification scheme of Vachaspati and Warnow [43]. We then added a single reticulation to each random model tree using the following procedure: (1) choose a random time unit *t*_*M*_ such that $0.01 \leq t_{M} \leq \frac {h}{4}$, and (2) add unidirectional migration, with a rate of 5.0 between two taxa or subpopulations such that migration occurs from *t*_*M*_−0.01 to *t*_*M*_+0.01. A single outgroup was added for each model network at coalescent time 1.5*h*.

**Simulation of coalescent histories and gene trees.** We simulated 1000 gene trees for each random model network using ms [45]. The following ms command was used:



The -T parameter outputs the gene trees that represent the history of the sampled taxa. The -I parameter is followed by *k* that represents the number of populations. The list of integers (*n*_1_ *n*_2_ … *n*_*k*_) represents the number of taxa sampled for each population. We sampled one taxon per population. The -ej parameter specifies to move all lineages in population *i* to population *j* at time *t*_0_. The first -em parameter sets migration at time *t*_1_ from population *j* to population *i* to 5.0. The second -em parameter sets migration at time *t*_2_ from population *j* to population *i* to zero.

**Simulation of sequence evolution.** The gene trees output by ms were used as input to seq-gen [46], a sequence evolution program, which can simulate the evolution of sequences according to a finite-sites model. For each local genealogy simulated by ms, we simulated DNA sequence evolution using the Jukes-Cantor mutation model [47]. The total length of the simulated sequences was 1000 kb distributed equally across all the local genealogies (1000 bp per local genealogy). The following command was used to simulate the evolution of sequences:



The -mHKY parameter specifies the Jukes-Cantor mutation model. The -s parameter specifies mutation rate *θ* of 0.02, 0.04, 0.08, 0.16, 0.32, or 0.64. The -l parameter specifies the length of a sequence in base pairs.

**Replicates.** Each model condition in our study consisted of a distinct set of choices for the simulation parameters listed above. For each model condition, the simulation procedure was repeated to obtain twenty replicates.

**Multi-locus methods for phylogenetic network inference.** A single pipeline with two stages was used to infer a species phylogeny. The first stage consists of obtaining gene trees, where either true gene trees were used or FastTree was used to infer gene trees using the sequence alignments for the loci. The second stage uses the gene trees from the first stage to infer a species phylogeny.

To obtain estimated gene trees in the first stage, FastTree [48, 49] under the Jukes-Cantor model was used to infer the maximum-likelihood unrooted gene tree for each sequence alignment generated by seq-gen. Using a custom script, we converted the branch lengths from expected number of substitutions to coalescent time using equation (3.1) in [50]. We applied two techniques to root the gene trees. The first technique (two-step rooting) involves rooting the gene trees based on an outgroup using PAUP* [51]. Each unrooted gene tree was used as a backbone and the outgroup was added to root each gene tree under the maximum-likelihood criterion. After rooting each inferred gene tree, the outgroup taxon and its pendant edge were pruned. The second technique (one-step rooting) involves including the outgroup in the local gene tree inference using FastTree, and then rooting the maximum-likelihood unrooted gene tree generated by FastTree using the outgroup. The outgroup taxon and its pendant edge are then dropped.

In the second stage, the gene trees from the first stage were used as input to the following phylogenomic inference methods: MLE-length, MLE, MPL, MP, and SNaQ. MLE-length, MLE, MPL, and MP are implemented as part of the PhyloNet package [22]. The following is a sample NEXUS script file that was used to execute the PhyloNet commands:



The commands located in the TREES block contain the gene trees. The commands located in the PHYLONET block contain the inference methods and parameters used to infer a species network. The InferNetwork_ML command infers a species network with one reticulation node using maximum likelihood. The -bl parameter specifies the use of branch lengths of gene trees in the inference. In the absence of -bl, only the topologies of gene trees are used in the inference. The InferNetwork_MPL command infers a species network with one reticulation node using maximum pseudo-likelihood. The InferNetwork_MP command infers a species network with one reticulation node using a parsimony-based method under the MDC criterion.

The following is a sample script used to execute the SNaQ commands:



The gene trees are summarized as quartet concordance factors using the readTrees2CF function. The readTopology reads the tree used as a starting point for the search. The starting tree was estimated using the MDC criterion. The snaq! command estimates a network using the input quartet concordance factors *T* and starting from tree *d*. *hmax* specifies the number of reticulation nodes. *outgroup* specifies the outgroup taxon used to root the inferred network.

**Concatenation methods for phylogenetic network inference.** For the concatenation analyses, we inferred species networks using two distance-based methods implemented in the phangorn software package [52]: (1) Neighbor-Net [11], a clustering method that extends the neighbor-joining algorithm, and (2) the least squares method of Schliep [12]. Throughout this manuscript, we refer to the latter as SplitsNet. The LogDet distance [53] was used to calculate a distance matrix for use as input to the distance-based concatenation methods.

**Performance measures.** We evaluated the inference methods based upon their topological accuracy and computational requirements. Topological accuracy was evaluated by comparing the inferred phylogeny to the model phylogeny using the tripartition distance metric [54], which finds the proportion of tripartitions that are not shared between the model and inferred networks. We compare trees using the normalized Robinson-Foulds (RF) distance [55]. The RF distance counts the number of false positive bipartitions (bipartitions found in the inferred network but not the model network) and false negative bipartitions (bipartitions found in the model network but not the inferred network). Finally, we used the splits distance, which measures the proportion of bipartitions found in the trees encoded by the inferred species network but not in the trees encoded by the true species network and vice versa. The second evaluation criterion used was the computational requirements of the inference methods, which was measured in terms of CPU runtime and memory usage. Each analysis was run on a 2.5 GHz Intel Xeon E5-2670v2 processor with 128 GiB of main memory.

### Empirical study

We used genomic sequence data sampled from natural mouse populations. A recent study has highlighted historical gene flow between some of the populations in our study [3]. The samples were collected in previous studies [3, 56–60]. The collected sample information contained 100 haploid mouse genomes that are either wild or wild-derived samples. The procedure that was used to generate the sequence data is described in the study of Liu et al. [3]. The sequences were filtered to 414,376 SNPs that were genotyped across all samples.

Datasets were constructed from the empirical samples using the following sampling procedure. For each dataset, we randomly selected one sample from each of the following mouse species or subspecies: *Mus musculus domesticus*, *M. musculus musculus*, *M. musculus castaneus*, *M. spretus*, *M. spicilegus*, and *M. macedonicus*. The sampling was repeated twenty times to obtain twenty datasets.

We estimated recombination-free intervals for use as the input loci to the phylogenetic network inference methods. This required inferring recombination breakpoints. We obtained breakpoints using RecHMM, a hidden Markov model-based method [61], resulting in 3013 recombination-free genomic regions. For each recombination-free genomic region, FastTree was used to infer a gene tree with maximum likelihood under the Generalized Time-Reversible model [62], resulting in 3013 gene trees. We used rat (the rn5 assembly downloaded from the UCSC Genome Browser [63]) as an outgroup to root each gene tree generated by FastTree.

MLE-length, MP, and SNaQ were used to infer species networks with zero or one reticulation nodes. For inferred networks with zero reticulation nodes, we measured the topological distance between inferred trees using the Robinson-Foulds distance. For inferred networks with one reticulation node, the tripartition distance was used to compute the topological distance between inferred networks. We further compared the reticulations inferred by the inference methods to previous studies which detected two cases of gene flow: one among the *M. musculus* subspecies [56], and the other between *M. musculus domesticus* and *M. spretus* [3]. Inference accuracy was evaluated by computing the proportion of replicates for which the inferred phylogeny was consistent with either of the two known instances of gene flow. Finally, we compared the inferred networks to the consensus *Mus* phylogeny proposed by Guénet and Bonhomme [64]. Dendroscope [44] was used to visualize the empirical phylogenies.

## Results

### Performance evaluation on simulated datasets

**Fig. 1:**
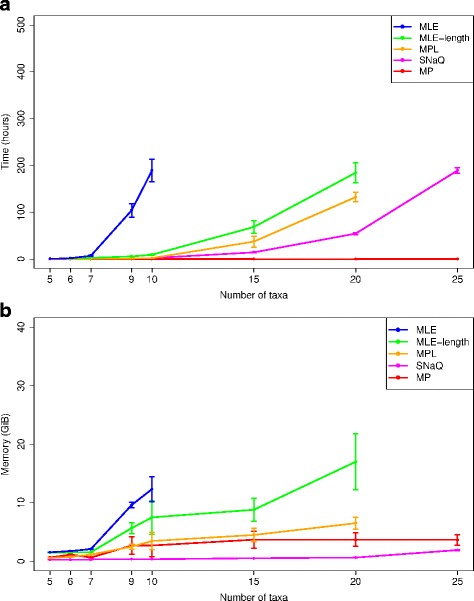
The impact of dataset size on the computational requirements of multi-locus methods. The model conditions had dataset sizes ranging from 5 to 25 taxa. Results are shown for MLE, MLE-length, MPL, MP, and SNaQ analyses using true gene trees as input. **a** Average runtime (h) and **b** main memory usage (GiB) are shown with standard error bars (*n*=20). The analysis of MLE on 15 taxa, MLE-length on 25 taxa, and MPL on 25 taxa did not complete after ten days of runtime

**Runtime and memory usage.** We began by assessing the effect of dataset size on computational time and memory requirements. Figure 1 shows results for the multi-locus methods, which were the most accurate methods in our study (see Additional file 1: Figure S1 for comparisons of the multi-locus methods and the concatenation methods).

For all methods except MP, runtime became impractical on datasets with more than a few dozen taxa. Of the full likelihood methods, MLE-length was consistently faster than MLE; the comparison of pseudo-likelihood-based methods revealed that SNaQ was consistently faster than MPL. Overall, we found that SNaQ was the fastest probabilistic multi-locus method. Given a maximum runtime of ten days, the fastest full likelihood method (MLE-length) and pseudo-likelihood-based method (SNaQ) were able to analyze datasets with 20 and 25 taxa, respectively. However, MLE-length and SNaQ required more than ten days of runtime on datasets with 25 and 30 taxa, respectively. The other multi-locus methods were slower: after ten days of runtime, MLE did not complete analyses of datasets with 15 taxa and MPL analyses did not complete on datasets with 25 taxa. We also attempted analyses of datasets with 40, 50 and 100 taxa; none of these analyses (MLE, MLE-length, MPL, and SNaQ) had finished after ten days of runtime and, in fact, are still running as of this writing after several months of CPU runtime. For the dataset sizes shown in Fig. 1(a), the full likelihood methods (MLE and MLE-length) had runtime and memory usage that were strictly greater than the pseudo-likelihood-based methods (MPL and SNaQ). For all methods except MP, runtime grew super-linearly as dataset size increased. The observed growth in runtime is similar to previous performance studies [25, 65, 66], which suggest an increase in runtime as sampled dataset sizes grow.

Relative to runtime performance, the main memory requirements of the different methods contrasted to a greater degree. On datasets with more than seven taxa, the full likelihood methods exhibited a super-linear growth in main memory usage, similar to its performance in terms of runtime (panel b in Fig. 1). The full likelihood methods’ main memory requirements are projected to become prohibitive on datasets with more than a few dozen taxa. MLE-length generally had lower memory requirements than MLE. In contrast, MP, MPL, and SNaQ had memory usage that was largely constant at around a few GiB on datasets with up to 25 taxa. MP and MPL had memory usage below 5 GiB on datasets with up to 20 taxa. SNaQ’s memory usage was flat at around 1 GiB as dataset size increased from 5 to 20 taxa, and increased by just a few GiB as dataset size increased from 20 to 25 taxa. Overall, SNaQ’s memory usage was the smallest among all multi-locus methods across all of the dataset sizes in our study.

**Topological accuracy.** We next examined the topological accuracy of the inference methods as dataset scale grew in two ways: the number of taxa and sequence divergence. We evaluated the topological accuracy of the inferred phylogenetic networks using tripartition distance [67] (see “Methods”).

**Fig. 2:**
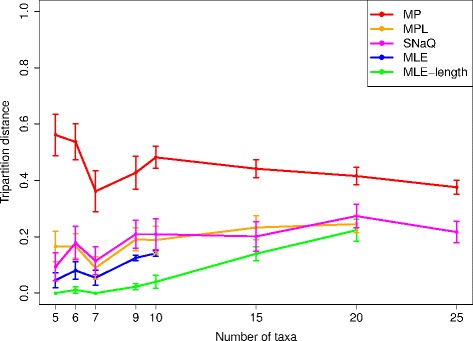
The impact of dataset size on the topological accuracy of multi-locus methods. The model conditions had dataset sizes ranging from 5 to 25 taxa. Results are shown for MP, MLE, MLE-length, MPL, and SNaQ using true gene trees as input. The tripartition distance between an inferred network and the model network was used to measure topological accuracy. Average distance and standard error bars are shown (*n*=20)

On sufficiently small datasets where analyses terminated, the probabilistic multi-locus methods returned improved topological accuracy compared to the parsimony-based multi-locus method (Fig. 2). Furthermore, the full likelihood methods (MLE-length and MLE) were more accurate than the pseudo-likelihood-based methods. Thus, the methods fell into four categories based upon topological accuracy. Across all model conditions, (1) MLE-length was the most accurate, (2) MLE was the second most accurate, (3) SNaQ and MPL were the third most accurate, and (3) MP was the least accurate method. Note that, for each replicate, the same set of gene trees was provided to each multi-locus method as input.

Overall, topological accuracy degraded as the number of taxa increased (Fig. 2 and Additional file 1: Figures S2 and S3). The accuracy of each method was generally smallest on the largest datasets in our study. Three exceptions to this observation were noted: (1) MP was consistently less accurate than the other methods but otherwise did not show a clear trend of change as dataset size increased, (2) MLE, MLE-length, MPL, and SNaQ’s topological accuracy decreased as dataset size increased from 6 to 7 taxa, and (3) SNaQ’s topological accuracy decreased as dataset size increased from 20 to 25 taxa.

**Fig. 3:**
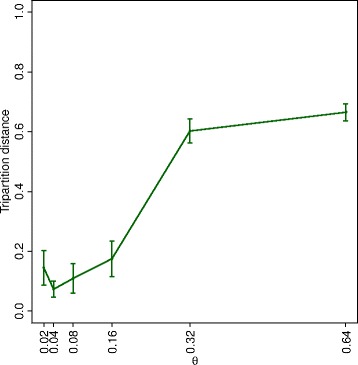
The impact of mutation rate on the topological accuracy of MLE-length. We assessed the performance of MLE-length to characterize the accuracy of multi-locus inference methods since MLE-length was generally more accurate than MLE, SNaQ, MPL, and MP (Fig. 2). The seven-taxon model conditions had mutation rate *θ* ranging from 0.02 to 0.64. The tripartition distance between an inferred network and the model network was used to measure topological accuracy. Average distance and standard error bars are shown (*n*=20)

We further examined the topological accuracy of the most accurate multi-locus inference method (MLE-length) as sequence divergence grew. We observed that the topological accuracy of MLE-length generally degraded as sequence divergence increased due to larger mutation rate *θ* (Fig. 3), with the exception of a relatively small improvement in accuracy as *θ* increased from 0.02 to 0.04. Compared to the rest of our simulation study, the seven-taxon model condition with mutation rate *θ*=0.64 was unique because it returned the highest topological error observed in our simulation study.

### Performance evaluation on empirical datasets

Our performance study utilized empirical samples from natural populations of *Mus musculus* subspecies and sister species (*M. spretus*, *M. spicilegus*, and *M. macedonicus*). Prior studies detected gene flow between the *M. musculus* subspecies [56] and between *M. musculus domesticus* and *M. spretus* [58, 68]. We focused our comparison on the most accurate methods from each category of multi-locus methods: MLE-length from the full likelihood methods, SNaQ from the pseudo-likelihood-based methods, and MP. We omitted the concatenation methods from our comparison since they were among the least accurate of all methods in our simulation study (see Additional file 1: Figure S1).

At a coarse level, probabilistic inference using MLE-length was able to accurately detect gene flow in the empirical datasets. Specifically, the model selection criterion used by MLE-length consistently chose solutions with gene flow (i.e., phylogenetic networks with one reticulation node) as opposed to solutions without gene flow (i.e., phylogenetic trees).

As shown in Table 1, all of the methods inferred an identical species tree topology when constrained to infer a solution involving zero reticulations. For the inferred phylogenetic networks, greater topological similarity was observed among phylogenies inferred using the same method as opposed to phylogenies inferred using different methods. Furthermore, greater topological agreement was observed when solutions were constrained to have no gene flow, as opposed to solutions involving gene flow. Based on intra-method comparison of inferred networks, the greatest topological agreement was observed among MLE-length, followed by SNaQ, and then MP. Topological comparison of the different methods (i.e., MLE-length compared to MP, MLE-length compared to SNaQ, and MP compared to SNaQ) yielded topological distances which were the highest observed in our empirical study, and comparable disagreement was observed between the different pairs of methods as measured by average topological distance.

**Table 1:** Topological distances between inferred phylogenies in the empirical study

Average (SE) topological distance between inferred phylogenetic						
networks						
	MLE-length		MP		SNaQ	
MLE-length	.11	(.02)	.42	(.06)	.44	(.04)
MP			.36	(.03)	.52	(.05)
SNaQ					.23	(.02)

Phylogenies were inferred using a representative method from each category of multi-locus methods: MLE-length (a full likelihood probabilistic method), MP (a parsimony-based method), and SNaQ (a pseudo-likelihood-based probabilistic method). The normalized tripartition distance between solutions that included gene flow (i.e., phylogenetic networks with one reticulation) is shown as an average (standard error) across replicates (*n*=20). When constrained to infer a phylogenetic tree rather than a phylogenetic network, all methods inferred an identical species tree across all replicates. Each replicate dataset consists of randomly selecting a sample from the following mouse species and subspecies: *Mus musculus domesticus*, *M. musculus musculus*, *M. musculus castaneus*, *M. spretus*, *M. spicilegus*, and *M. macedonicus*

We further evaluated whether the methods detected known instances of interspecific and intersubspecific gene flow: the former involving gene flow between *M. musculus domesticus* and *M. spretus* and the latter involving gene flow between the *M. musculus* subspecies. MP, SNaQ, and MLE-length inferred a phylogenetic network consistent with gene flow between *M. musculus domesticus* and *M. spretus* in 12, 0, and 15 replicates, respectively (out of 20 replicates in total); the three methods inferred a network consistent with intersubspecific gene flow among the *M. musculus* subspecies in 0, 17, and 3 replicates, respectively.

We also compared the phylogenetic networks inferred by MLE-length, SNaQ, and MP to a consensus *Mus* species tree obtained from prior literature studies [64]. (The consensus tree is visualized in Additional file 1: Figure S5.) The bipartitions in the consensus tree were consistently inferred by the different methods. Compared to MP, the probabilistic multi-locus methods more frequently inferred reticulations that were consistent with known interspecific/intersubspecific gene flow; however, the methods largely disagreed on the exact location of reticulation within the phylogeny.

## Discussion

The probabilistic multi-locus methods were the most accurate methods in our study, but they were also among the most computationally intensive methods in terms of runtime. SNaQ was the fastest probabilistic multi-locus method in our study, but was not generally more accurate than the full likelihood methods; our findings are consistent with the observations of Solís-Lemus and Ané [25]. However, given ten days of runtime, none of these methods completed analyses of datasets with more than two dozen taxa. Our finding resolves an open question in the literature: can state-of-the-art phylogenetic network inference methods scale to dataset sizes typically seen in today’s phylogenomic studies? Our study has shown that the answer is no, despite the methodological tradeoffs made by some of the methods. For example, the pseudo-likelihood-based methods use an approximation to full likelihood calculations under the coalescent model to improve scalability. Based on the discussion in the study of Solís-Lemus and Ané [25], we expected that the tradeoff would yield at least several factors of runtime improvement compared to full likelihood methods, at the cost of reduced topological accuracy. Instead, the tradeoff only scaled up analyses of around 20 to 25 taxa. On datasets with more than 30 taxa, the computational requirements of the probabilistic multi-locus methods are projected to be nearing the limits of the most powerful computational clusters available to us. This dataset size is the largest in our study and yet is not considered large in the context of today’s phylogenomic studies. We expect that, like the full likelihood method, the pseudo-likelihood-based methods’ memory requirements will grow super-linearly as dataset sizes increase past an inflection point. Finding the inflection point will require additional experiments using dataset sizes larger than those explored in our study.

Our study generally shows that the performance comparison between the different classes of methods holds as dataset size and divergence increases, and further quantifies the impact upon topological accuracy. Increasing either of the two dimensions of scale – the number of taxa and sequence divergence – generally reduced the topological accuracy of each method. Both observations are consistent with related studies of phylogenetic tree inference in the presence of gene flow [34, 35]; the studies also suggest other factors impacting scalability (e.g., number of loci). The heuristic approaches necessary for analysis of NP-hard optimization problems contribute to the methods’ scalability; practical issues such as local optima in the search space can pose major challenges to the performance of these heuristics. We observed a U-shaped trend where topological accuracy improved slightly as the mutation rate *θ* increased from 0.02 to 0.04, and accuracy rapidly degraded as the mutation rate increased past 0.04. The trend is likely due to two factors. First, we conjecture that the model condition with the smallest mutation rate in our study had relatively low sequence variation and therefore offered little phylogenetic signal. Second, increased sequence divergence due to increasing mutation rates reduced the accuracy of inferred gene trees, which is consistent with theoretical expectations and empirical observations about long branch attraction in other phylogenetic studies [69]. The inferred gene tree error observed in our study (Additional file 1: Table S2) was comparable to that of other performance studies [70, 71], ranging from 0.38 to 0.80 as *θ* grew from 0.02 to 0.64 for the seven taxon model.

Our study included probabilistic multi-locus methods that either accounted for or ignored branch length information in input gene trees. In particular, MLE-length and MLE were identical methods with one major exception: the former calculated model likelihood using gene tree topologies and branch lengths [27], whereas the latter substituted the approach of Degnan and Salter [23] which calculates model likelihood using only gene tree topologies. We found that MLE-length returned greater topological accuracy than MLE, which is consistent with the prevailing opinion that models incorporating branch length information will be generally more accurate than inference under related models that ignore branch length information [27] (although see the review of Nakhleh [10] for an opposing viewpoint). The pseudo-likelihood-based methods (MPL and SNaQ) were less accurate than the full likelihood methods. This is expected as pseudo-likelihood-based methods were designed to tradeoff inference accuracy for computational efficiency. Consistent with other performance studies examining the related problem of scalable phylogenetic tree estimation [17, 69, 72], the parsimony-based multi-locus method were not as accurate as the most accurate probabilistic multi-locus method. The topological accuracy of MP improved somewhat as dataset size increased from 5 to 25 taxa. We attribute this finding to long branch attraction, where increasing numbers of taxa and constant model phylogeny height resulted in denser taxon sampling. The impact of long branches on parsimony-based phylogenetic inference is well understood [72], and may cause it to be more vulnerable to long branch attraction issues compared to the other probabilistic methods in our study. We also compared the most accurate multi-locus inference method (MLE-length) to two concatenation methods (SplitsNet and Neighbor-Net). The concatenation methods were less accurate than MLE-length as dataset size increased from 5 to 10 taxa. These results are supported by other performance studies focusing on the problem of phylogenetic tree estimation [17].

Using an information theoretic approach for model selection, MLE-length consistently inferred historical gene flow between the sampled mouse populations in our study, which is consistent with prior studies [56, 68]. MLE-length was the most accurate method in terms of detecting gene flow supported by literature evidence. Similarly, MLE-length was the only tested inference method that detected the two gene flow events supported by the literature (i.e. gene flow between the *M. musculus* subspecies and between *M. musculus domesticus* and *M. spretus*). SNaQ was the second most accurate method, and detected gene flow among the *M. musculus* subspecies but not between *M. musculus domesticus* and *M. spretus*. MP was least accurate and detected gene flow between *M. musculus domesticus* and *M. spretus* but not among the *M. musculus* subspecies. These findings support the observations in our simulation study, where full likelihood-based methods returned improved accuracy compared with pseudo-likelihood-based and parsimony-based methods. For inferred networks, none of the methods were robust to the choice of sampled taxa. This suggests low support which could be due to several causes, including the impact of dataset size and sequence divergence on inference error (consistent with the simulation study) and/or a soft polytomy due to a short branch involving *M. musculus* subspecies (consistent with the consensus phylogeny proposed by Guénet and Bonhomme [64]). Furthermore, except for MP, the topological distances observed in the empirical study were larger than the topological errors observed in comparable datasets from our simulation study. Even assuming that a species phylogeny inferred on one of the replicates was correct (or close to correct), the topological distances between inferred phylogenies imply that inferences on many of the other replicates would have error comparable to or greater than those observed in the simulation study. One contributing factor is that the empirical datasets may pose a more difficult inference problem since they reflect a broader array of evolutionary processes than those involved in the simulation study. For example, positive selection and recombination have been shown to play significant roles in the evolution of the natural house mouse populations that were sampled in our study [56, 58, 68].

## Conclusions

In this study, we have evaluated the scalability of state-of-the-art methods for inferring phylogenetic networks from multi-locus sequences that evolved under genetic drift/ILS, gene flow, and point mutations, where much of the difficulty of this inference problem is due to the complex interplay of all three evolutionary processes. (The absence of drift and ILS would imply that a unique solution can be found in a straightforward manner for the model phylogenies considered in our study). We quantified the performance of the methods in terms of computational runtime, main memory usage, and topological accuracy on datasets that varied along two separate dimensions of scale: the number of taxa and sequence divergence.

The methods in our study face tremendous scalability challenges on datasets that are well within the scope of today’s phylogenomic studies. In terms of accuracy, the probabilistic multi-locus methods outperformed the other methods, which is consistent with the state of the art of phylogenetic tree inference. For this reason, we generally recommend using the former – particularly MLE or MLE-length, a full likelihood-based approach – rather than the latter. The latter included concatenation methods – the predominant approach used in today’s phylogenomic studies. More taxa and greater sequence divergence degraded the topological accuracy of all methods. While the probabilistic multi-locus methods retained a performance advantage in terms of topological accuracy, their computational requirements were excessive. On datasets with fewer than 25 taxa, the pseudo-likelihood-based multi-locus method (SNaQ) generally completed analysis within a day using around a few GiB of main memory or less; the full likelihood multi-locus method (MLE-length) was able to analyze datasets with around 15 taxa within a day and required around 10 GiB of main memory; the full likelihood multi-locus method (MLE) was able to analyze datasets with around 7 taxa within a day and required around 10 GiB of main memory. The computational requirements of the probabilistic multi-locus methods grew rapidly as the number of taxa increased and became prohibitive on datasets with more than 30 taxa. We note that, in a sense, the two dimensions of scale act in opposition: increasing taxon sampling can help reduce individual branch lengths and mitigate the negative effect of long branch attraction on inference accuracy, but comes at the cost of increasing the number of taxa which increases runtime requirements and can also increase inference error as well.

We highlight several aspects of our study for future work. Most importantly, our study has highlighted the clear need for new phylogenetic inference methods that can cope with the scale of current phylogenomic studies, involving as many as hundreds of genomes; the near future will bring studies that are orders of magnitude larger. We anticipate that our study foreshadows new methodological development on the topic of large-scale phylogenetic network inference. An expanded empirical study with larger datasets will be possible as future studies follow up on initial reports of gene flow among natural populations (particularly involving different species) and perform additional sequencing. Finally, we propose that the dichotomy between the different categories of methods in our study represents an algorithmic engineering opportunity. By synthesizing these approaches, advantages in one category of methods can help offset disadvantages in the other.

## Additional file

Additional file 1 Appendix with Supplementary Material. Appendix, including text, tables, and figures for supplementary experiments. (PDF 292 kb)

## Abbreviations

HMM: Hidden Markov model
ILS: Incomplete lineage sorting
MDC: Minimize deep coalescence
MLE: Maximum likelihood estimation
MPL: Maximum pseudo-likelihood
MP: Maximum parsimony
RF distance: Robinson-Foulds distance
SNaQ: Species networks applying quartets
