# Supplementary material for: A scalability study of phylogenetic network inference methods using empirical datasets and simulations involving a single reticulation
Source: BMC Bioinformatics. 2016 Oct 13;17:422. doi: 10.1186/s12859-016-1277-1 (PMC5064893; doi:10.1186/s12859-016-1277-1)
Supplement: Supplementary file 1 — Appendix with Supplementary Material. Appendix, including text, tables, and figures for supplementary experiments. (PDF 292 kb) [file 12859_2016_1277_MOESM1_ESM.pdf]

Supplementary Materials

Hussein A. Hejase & Kevin J. Liu

1 Comparison between multi-locus and concatenation methods

We compared the most accurate multi-locus inference method (MLE-length) to two concatenation methods which include NeighborNet [1] and the least squares method of Schliep [2], which we refer to here as SplitsNet. We ran the concatenation methods using their default settings. The splits distance was used to evaluate the topological error, which quantifies the proportion of bipartitions that differ between the model and inferred phylogenies. As shown in Figure S1, the three methods fell into three categories based on their topological accuracy: MLE-length was the most accurate, NeighborNet was the second most accurate, and SplitsNet was the least accurate method. These results suggest that concatenation methods are less accurate than multi-locus inference methods. We also observed an increase in the topological error across all methods as the number of taxa increased from five to ten.

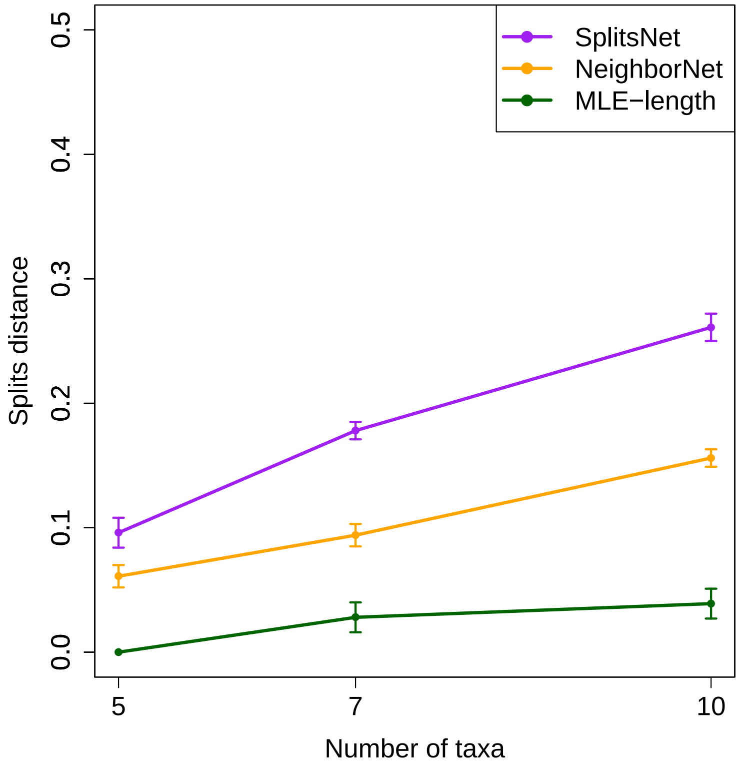

Figure S1: **The impact of number of taxa on the topological accuracy of MLE-length and concatenation methods (SplitsNet and NeighborNet).** We assessed the performance of MLE-length to characterize the accuracy of multi-locus inference methods since MLE-length was generally more accurate than MLE, MPL, SNaQ, and MP on model conditions with true gene trees. The three model conditions had dataset sizes ranging from 5 to 10 taxa and a mutation rate θ of 0.08. The splits distance between an inferred network and the model network was used to measure topological accuracy. Average distance and standard error bars are shown ($n = 20$).

2 Rooting techniques

We utilized two approaches to root gene trees inferred by FastNet. The first rooting technique (one-step rooting) involves including the outgroup in the gene tree inference, and then roots the inferred gene trees using the outgroup. Finally, the outgroup taxon and its pendant edge are dropped. The second rooting technique (two-step rooting) involves running FastTree with no outgroup. The next step involves adding the outgroup to the unrooted gene trees using PAUP* [3]. The unrooted gene trees were used as a backbone and then the outgroup was used to root the gene trees under the maximum-likelihood criterion. Finally, the outgroup taxon and its pendant edge are dropped. As show in Figure S2, we observed no significant accuracy difference between both rooting techniques.

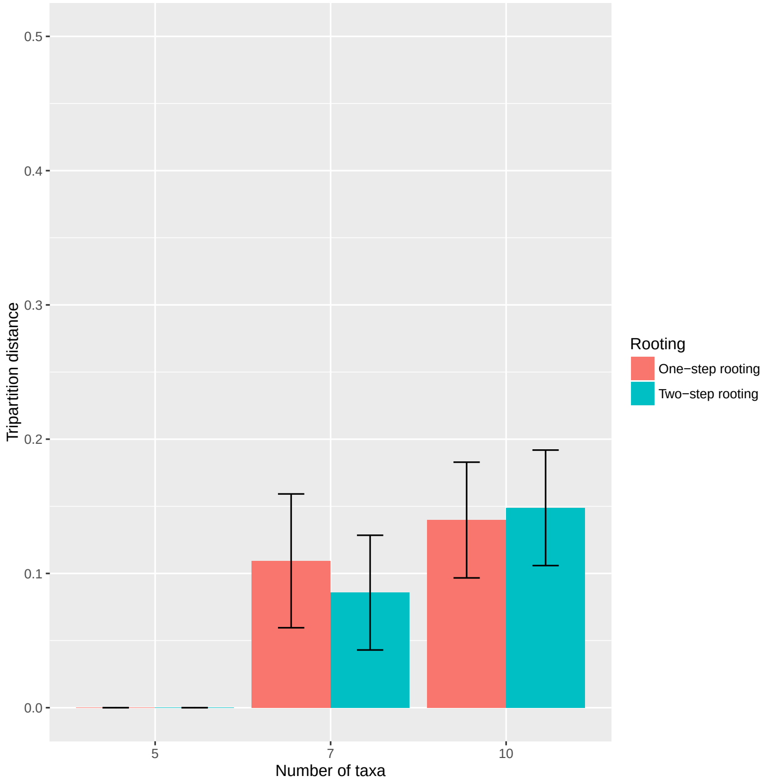

Figure S2: **The impact of two rooting techniques on the topological accuracy of MLE-length.** We assessed the performance of MLE-length to characterize the accuracy of rooting techniques since MLE-length was generally more accurate than MLE, MPL, SNaQ, and MP on model conditions with true gene trees. The three model conditions had dataset sizes ranging from 5 to 10 taxa and a mutation rate θ of 0.08. The tripartition distance between an inferred network and the model network was used to measure topological accuracy. Average distance and standard error bars are shown ($n = 20$).

3 Performance of MLE-length using inferred gene trees

We evaluated the performance of the most accurate multi-locus inference method (MLE-length) as dataset size increased. The topological error of MLE-length increased as the number of taxa increased from five to ten.

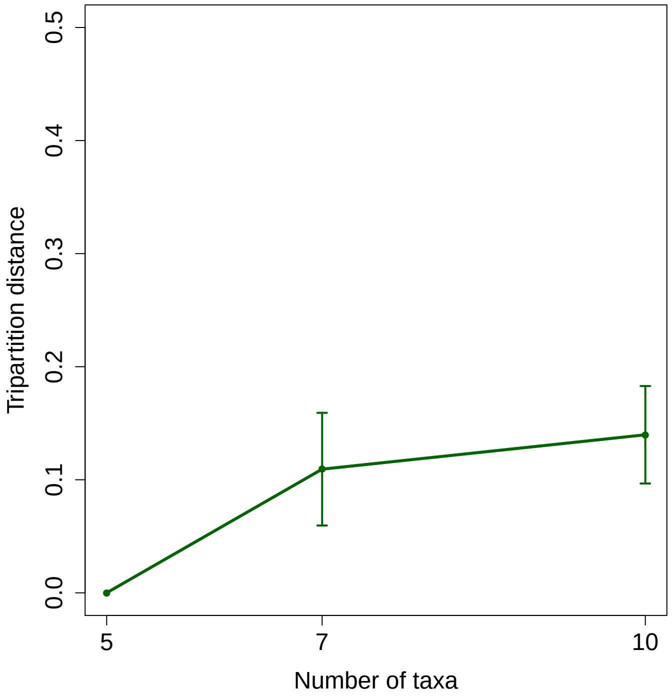

Figure S3: **Topological accuracy of MLE-length using inferred gene trees instead of true gene trees.** We assessed the performance of MLE-length to characterize the accuracy of multi-locus inference methods since MLE-length was generally more accurate than MLE, MPL, SNaQ, and MP on model conditions with true gene trees. The model conditions had dataset sizes ranging from 5 to 10 taxa and a mutation rate θ of 0.08. The tripartition distance between an inferred network and the model network was used to measure topological accuracy. Average distance and standard error bars are shown ($n = 20$).

4 Gene tree error

We measured the topological error between true gene trees using Robinson-Foulds (RF) distance. Table S1 reports the RF distance between true gene trees for dataset sizes ranging from 5 to 25 taxa using simulated data. On average, there is around 76.9% disagreement between true gene trees. Table S2 reports the RF distance between inferred gene trees for 5, 7, and 10 taxa across different θ values. On average, as θ increases from 0.02 to 0.64, the average RF distance increases from 0.38 to 0.8, respectively. This suggests that as sequence divergence increases, the topological error of the inferred gene trees increases accordingly. We did not observe a trend in the topological error as the number of taxa for θ value of 0.08 increased from 5 to 9.

**Number of taxa**	**Average ± SE**
**5**	0.70 ± 0.02
**6**	0.71 ± 0.01
**7**	0.72 ± 0.01
**9**	0.77 ± 0.01
**10**	0.77 ± 0.01
**15**	0.81 ± 0.01
**20**	0.82 ± 0.01
**25**	0.85 ± 0.01

Table S1: **Mean and standard error across 20 replicates of the RF distance between true gene trees for dataset sizes ranging from 5 to 25 taxa.**

**Number of taxa**	θ**=0.02**	θ**=0.04**	θ**=0.08**	θ**=0.16**	θ**=0.32**	θ**=0.64**
**5**	NA	NA	0.31 ± 0.01	NA	NA	NA
**7**	0.38 ± 0.03	0.38 ± 0.03	0.40 ± 0.03	0.49 ± 0.03	0.68 ± 0.02	0.80 ± 0.01
**10**	NA	NA	0.34 ± 0.02	NA	NA	NA

Table S2: **Mean and standard error across 20 replicates of the RF distance between inferred gene trees for θ values ranging from 0.02 to 0.64.**

5 Visualization of inferred and model networks

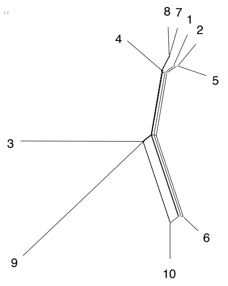

(a) Inferred phylogeny using NeighborNet

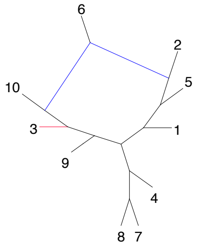

(b) Inferred phylogeny using MLE-length

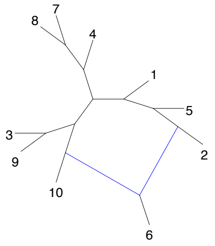

(c) Model phylogeny

Figure S4: **Visualization of NeighborNet, MLE-length, and the model phylogenies.** Results are shown for an example replicate for ten taxa using inferred gene trees and a mutation rate θ of 0.08. We assessed the performance of MLE-length and NeighborNet to characterize the accuracy of multi-locus and concatenation inference methods since they were generally the most accurate methods in their respective categories. (a) The inferred phylogeny by NeighborNet, which was run using its default settings. (b) The inferred phylogeny by MLE-length. (c) The model phylogeny generated by ms. The blue lines in (b) and (c) represent the reticulation edges. The red line in (b) represents the incorrectly inferred edge by MLE-length.

6 Empirical consensus phylogeny

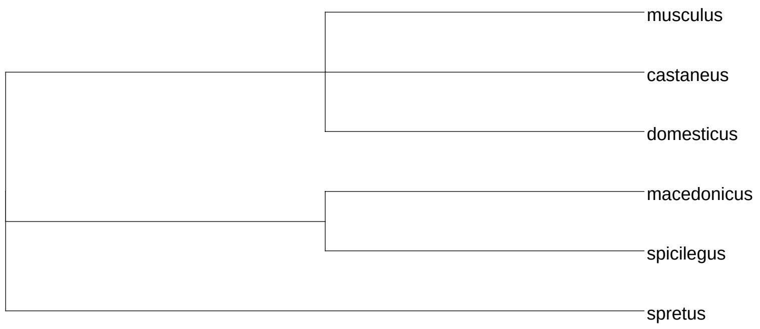

Figure S5: **The *Mus* consensus phylogeny proposed by Guénet and Bonhomme [4].** Previous studies [5, 6] identified gene flow between the *M. musculus* subspecies and between *M. musculus domesticus* and *M. spretus*.

7 Empirical data

Sample name	Type (Origin)
B9	Wild caught (Hamm, North Rhine-Westphalia, Germany)
B10	Wild caught (Hamm, North Rhine-Westphalia, Germany)
B11	Wild caught (Hamm, North Rhine-Westphalia, Germany)
C1	Wild caught (Hamm, North Rhine-Westphalia, Germany)
C2	Wild caught (Hamm, North Rhine-Westphalia, Germany)
C3	Wild caught (Hamm, North Rhine-Westphalia, Germany)
MWN1287	Wild caught (Roca del Valles, Catalunya, Spain)
PERC/EiJ	Wild-derived laboratory strain (Nana Village, Rimac Valley, Peru)
WSB/EiJ	Wild-derived laboratory strain (Centerville, Maryland, US)
ZALENDE/EiJ	Wild-derived laboratory strain (Zalende, Switzerland)
MWN1279	Wild caught (Arel, Mallorca island, Spain)
RDS12763	Wild caught (Tubingen, Germany)
KCT222	Wild caught (Remderoda, Germany)
MWN1194	Wild caught (Korinthos, Velo, Peleponissos, Greece)
MWN1198	Wild caught (Laganas, Zakinthos Island, Greece)
MWN1026	Wild caught (San Girogio, Curone Valley, Piamonte, Italy)
MWN1030	Wild caught (Menconico, Staffora Valley, Lombardia, Italy)
MWN1106	Wild caught (Cassino, Lazio, Italy)
MWN1214	Wild caught (Milazzo, Olivarella, Sicily, taly)
22MO	Wild-derived laboratory strain (Monastir, Tunisia)
WMP	Wild-derived laboratory strain (Monastir, Tunisia)
DMZ	Wild-derived laboratory strain (Azemmour, Moroco)
BZO	Wild-derived laboratory strain (Oran, Algeria)
DCA	Wild-derived laboratory strain (Akrotiri, Cyprus)
DCP	Wild-derived laboratory strain (Paphos, Cyprus)
CZECHII/EiJ	Wild-derived laboratory strain (Bratislava, Slovak Republic)
PWK/PhJ	Wild-derived laboratory strain (Lhotka, Bohemia, Czech Repuplic)
SKIVE/EiJ	Wild-derived laboratory strain (Skive, Denmark)
BAG102	Wild caught (Gabortelep, Bekes, Hungary)
BAG3	Wild caught (Bukovce, Slovak Republic)
BAG56	Wild caught (Pomykow, Lublin, Poland)
BAG68	Wild caught (Wola Duza, Lublin, Poland)
BAG74	Wild caught (Krasne, Podkarpackie, Poland)
BAG94	Wild caught (Szepes, Debrecen, Hajdu-Bihar, Hungary)
BAG99	Wild caught (Szomolyom, Hajdu-Bihar, Hungary)
RDS10105	Wild caught (Monchhof, Austria)
RDS13554	Wild caught (Hubinger-Leitham, Austria)
Yu2097m	Wild caught (Urumqi, Xinjiang, China)
Yu2099f	Wild caught (Urumqi, Xinjiang, China)

Yu2115m	Wild caught (Yutian, Xinjiang, China)
Yu2120f	Wild caught (Hebukesaier, Xinjiang, China)
CIM1	Wild-derived laboratory strain (Masinagudi, India)
POHN	Wild-derived laboratory strain
CAST/EiJ	Wild-derived laboratory strain (Thonburi, Thailand)
SPRET/EiJ	Wild caught (Puerto Real, Cadiz Province, Spain)
SEG1	Inbred lab
ZRU1	Inbred lab
YCA1	Inbred lab
XBS1	Inbred lab

Table S3: **Empirical mice genomic data along with their type and origin (City, Province, Country).** Origin was only reported for the wild-derived and wild caught laboratory strains.

References

[1] Bryant, D., Moulton, V.: Neighbor-Net: an agglomerative method for the construction of phylogenetic networks. Molecular Biology and Evolution **21**(2), 255–265 (2004)

[2] Schliep, K.: Some applications of statistical phylogenetics. PhD thesis, Massey University (2009)

[3] Swofford, D.L.: PAUP*: Phylogenetic Analysis Using Parsimony (*and Other Methods), Version 4. Sinauer Associates, Sunderland, Massachusetts. (2003)

[4] Guénet, J.-L., Bonhomme, F.: Wild mice: an ever-increasing contribution to a popular mammalian model. Trends in Genetics **19**(1), 24–31 (2003). doi:10.1016/S0168-9525(02)00007-0

[5] Liu, K.J., Steinberg, E., Yozzo, A., Song, Y., Kohn, M.H., Nakhleh, L.: Interspecific introgressive origin of genomic diversity in the house mouse. Proceedings of the National Academy of Sciences **112**(1), 196–201 (2015)

[6] Staubach, F., Lorenc, A., Messer, P.W., Tang, K., Petrov, D.A., Tautz, D.: Genome patterns of selection and introgression of haplotypes in natural populations of the house mouse (*Mus musculus*). PLoS Genetics **8**(8), 1002891 (2012)
